# Prevalence of ABO Blood Groups and Their Relationship with Vascular Access Thrombosis and Mortality in Hemodialysis Patients

**DOI:** 10.3390/medicina62071227

**Published:** 2026-06-24

**Authors:** Can Hüzmeli, Ayşe Şeker, Hatice Ortaç, Nurettin Yeral

**Affiliations:** 1 Department of Nephrology, Hatay Training and Research Hospital, Hatay 31060, Türkiye; chuzmeli@hotmail.com; 2 Department of Nephrology, University of Health Sciences, Bursa City Hospital, Bursa 16009, Türkiye; 3 Department of Biostatistics, Institute of Health Sciences, Uludağ University, Bursa 16059, Türkiye; haticeortac21@gmail.com; 4 Department of Cardiology, Hatay Training and Research Hospital, Hatay 31060, Türkiye; dryeral@hotmail.com

**Keywords:** hemodialysis, ABO blood-group system, vascular access, thrombosis, mortality

## Abstract

*Background and Objectives:* ABO and Rh blood group systems represent clinically relevant genetic polymorphisms with established associations beyond transfusion medicine, including thrombotic risk. We investigated the prevalence of ABO and Rh blood group phenotypes in hemodialysis patients and their associations with documented vascular access thrombosis and all-cause mortality. *Materials and Methods:* This retrospective cohort study analyzed 3027 patients receiving maintenance hemodialysis in Hatay province, Türkiye, between January 2010 and April 2025. Data included ABO and Rh blood group determination, demographics, comorbidities, dialysis vintage, vascular access type, vascular access thrombosis events, and mortality. Multivariable binary logistic regression was used to identify independent factors associated with documented vascular access thrombosis. Multivariable Cox proportional hazards regression with vascular access thrombosis modeled as a time-dependent covariate was used to identify independent predictors of all-cause mortality. *Results:* Mean patient age was 63.95 ± 13.74 years; 58.3% were men. Blood group A was most prevalent (41.4%), followed by O (35.8%), B (15.9%), and AB (6.9%); 92.7% were Rh-positive. Documented vascular access thrombosis differed significantly by ABO group (*p* = 0.027), with the highest rate in group A (14.1%). In multivariable logistic regression, non-O blood group (OR 1.34, 95% CI 1.06–1.70; *p* = 0.014) and longer dialysis vintage (OR 1.01 per month, 95% CI 1.00–1.01; *p* < 0.001) were independently associated with documented vascular access thrombosis. In multivariable Cox regression, time-dependent vascular access thrombosis was independently associated with higher all-cause mortality (HR 1.33, 95% CI 1.12–1.59; *p* = 0.002), as were age (HR 1.02; *p* < 0.001), diabetes mellitus (HR 1.49; *p* < 0.001), coronary artery disease (HR 1.34; *p* < 0.001), and hypertension (HR 1.19; *p* < 0.001). Arteriovenous fistula was associated with lower mortality compared with temporary catheter (HR 0.46, 95% CI 0.37–0.58; *p* < 0.001). Blood group phenotype was not independently associated with all-cause mortality (all *p* > 0.5 vs. group O). *Conclusions:* In hemodialysis patients, non-O blood groups were modestly but independently associated with documented vascular access thrombosis, and vascular access thrombosis was independently associated with increased mortality when modeled as a time-dependent exposure. Blood group phenotype was not independently associated with mortality after adjustment for established risk factors. Blood group may contribute incrementally to vascular access risk awareness alongside established clinical risk factors, but its modest absolute risk difference limits standalone clinical utility.

## 1. Introduction

The ABO blood group system is one of the most clinically relevant genetic polymorphisms in humans. Beyond transfusion medicine and organ transplantation, ABO blood groups have been linked to cardiovascular, infectious, and neoplastic diseases [1,2,3,4]. Non-O individuals show elevated risks of venous thromboembolism, myocardial infarction, cerebrovascular events, and peripheral vascular disease compared with group O, largely attributable to higher plasma levels of von Willebrand factor (vWF) and factor VIII [4,5].

Patients on maintenance hemodialysis represent a unique population with complex alterations in hemostasis, inflammation, and cardiovascular risk. Vascular access complications, particularly thrombosis, remain a major source of morbidity, healthcare cost, and mortality in this population. Given the established link between ABO phenotype and thrombotic risk in the general population, examining this relationship in hemodialysis patients may inform risk awareness and clinical management.

The present study was designed to evaluate the prevalence of ABO and Rh blood group phenotypes in a large Turkish hemodialysis cohort and to examine their associations with documented vascular access thrombosis and long-term all-cause mortality, using methodology appropriate for time-to-event outcomes.

## 2. Materials and Methods

### 2.1. Study Design and Population

This retrospective cohort study analyzed patients receiving maintenance hemodialysis in Hatay province, Türkiye, between January 2010 and April 2025. Dialysis vintage was calculated from the date of first hemodialysis initiation regardless of when treatment began relative to the study timeframe.

### 2.2. Inclusion and Exclusion Criteria

Patients were eligible for inclusion if they (1) were aged 18 years or older and (2) had received hemodialysis treatment for a minimum of three months between January 2010 and April 2025. Exclusion criteria were (1) home hemodialysis and (2) absence of documented ABO and Rh blood group typing.

### 2.3. Data Collection

Patient data were systematically extracted from medical records, including demographic characteristics, primary renal diagnoses, dialysis vintage, vascular access type, comorbidities, and clinical outcomes. Vascular access type was categorized as arteriovenous fistula, permanent (tunnelled) catheter, or temporary catheter. Blood group (ABO and Rh) determinations were obtained from routine clinical laboratory testing performed as part of standard care, and classifications were verified against source laboratory records prior to analysis. Comorbidities were documented based on established diagnostic criteria, including diabetes mellitus, hypertension, coronary artery disease, and cerebrovascular disease. Vascular access-related thrombosis events were recorded from clinical documentation and interventional procedure reports. When available, the documented month of vascular access thrombosis was also recorded to allow time-dependent modelling in the mortality analysis.

### 2.4. Outcome Measures

The primary outcome was documented vascular access thrombosis during the follow-up period, defined as thrombosis recorded in medical records and/or interventional procedure reports. Because thrombosis events were identified retrospectively from documented clinical records rather than through standardized prospective surveillance, this outcome was interpreted as documented vascular access thrombosis and may underestimate the true incidence of thrombotic events. For patients with thrombosis, the documented month of thrombosis was used in time-dependent Cox regression modelling. Patients without documented thrombosis were considered free of thrombosis throughout the observed follow-up period. Secondary outcomes included all-cause mortality and the relationship between blood group phenotypes and baseline comorbidities. Survival data were collected through medical record review and confirmed through institutional databases.

### 2.5. Statistical Analysis

Statistical analyses were performed using SPSS version 25.0 (IBM Corp., Armonk, NY, USA). The normality of continuous variables was assessed using the Shapiro–Wilk test. Variables following a normal distribution were expressed as mean ± standard deviation, whereas non-normally distributed variables were presented as median (minimum–maximum). Categorical variables were reported as frequencies and percentages.

For between-group comparisons involving two groups with non-normal distributions, the Mann–Whitney U test was used. When comparing more than two groups with non-normal distributions, the Kruskal–Wallis test was applied, followed by post hoc analysis using the Dunn–Bonferroni test when overall significance was detected. Categorical variables were compared using Pearson’s chi-square test or Fisher’s exact test, as appropriate. Effect sizes were reported as Cramer’s V for categorical associations and epsilon-squared (ε^2^) for continuous variables, compared using the Kruskal–Wallis test.

Binary logistic regression analysis was performed to identify independent factors associated with documented vascular access thrombosis. In the multivariable logistic regression model, age, sex, dialysis vintage, diabetes mellitus, vascular access type, and blood group category were entered simultaneously using the enter method, based on clinical relevance rather than univariate *p*-value screening, to avoid model instability arising from correlated comorbidities. For this analysis, blood group was dichotomized as O versus non-O, with group O as the reference category, and vascular access type was included as a categorical covariate (reference: temporary catheter). Results were reported as odds ratios (ORs) with 95% confidence intervals (CIs). Model fit was assessed using the Hosmer–Lemeshow goodness-of-fit test, and overall model significance was evaluated.

Survival analysis was conducted using the Kaplan–Meier method to evaluate unadjusted differences in survival among ABO blood group categories, with comparisons performed using the log-rank test. Cox proportional hazards regression was used to identify independent predictors of all-cause mortality. Clinically relevant covariates, including age, sex, diabetes mellitus, hypertension, coronary artery disease, vascular access type, and blood group phenotype, were entered into the multivariable model simultaneously. Because vascular access thrombosis is a time-dependent event, it was not modeled as a fixed baseline covariate; instead, after the documented month of thrombosis became available, vascular access thrombosis was included in the Cox regression model as a time-dependent covariate. Patients were considered unexposed before the documented month of thrombosis and exposed thereafter. The proportional hazards assumption was verified through examination of Schoenfeld residuals. Results were reported as hazard ratios (HRs) with 95% CIs.

ABO–Rh phenotype-level subgroup analyses were considered exploratory. Because of the small sample sizes in some phenotype categories, these analyses were interpreted cautiously. No formal adjustment for multiple comparisons was applied to exploratory ABO–Rh phenotype-level subgroup analyses; therefore, these findings were interpreted descriptively and were not used as the basis for the main conclusions. A type I error rate of 5% (α = 0.05) was considered statistically significant for all main analyses.

## 3. Results

### 3.1. Study Population Characteristics

The study cohort comprised 3027 hemodialysis patients with a mean age of 63.95 ± 13.74 years (range 18–90 years; median 66 years). Male patients constituted 58.3% (*n* = 1766) of the population, while female patients represented 41.7% (*n* = 1261). The mean hemodialysis duration was 67.07 ± 54.80 months (range 3–360 months; median 53 months).

During the follow-up period, mortality occurred in 74.7% of patients (*n* = 2260), while 25.3% (*n* = 767) remained alive at study conclusion. Comorbidity analysis revealed diabetes mellitus in 54.9% of patients (*n* = 1647), hypertension in 53.0% (*n* = 1588), coronary artery disease in 21.8% (*n* = 659), and cerebrovascular disease in 7.7% (*n* = 231). Arteriovenous fistula was the predominant vascular access type (84.4%, *n* = 2555), followed by permanent (tunnelled) catheter (12.4%, *n* = 376) and temporary catheter (3.2%, *n* = 96). Documented vascular access thrombosis was identified in 12.3% of the cohort (*n* = 373).

### 3.2. ABO and Rh Blood Group Distribution

Blood group A demonstrated the highest prevalence at 41.4% of the study population, followed by group O (35.8%), group B (15.9%), and group AB (6.9%). Overall, 92.7% of patients were Rh-positive. Within each ABO group, the proportion of Rh-negative individuals ranged from 6.2% (group B) to 11.9% (group AB). When expressed relative to the entire cohort, the AB Rh-negative phenotype represented 0.83% (25/3027), and the rarest phenotypes corresponded to expected population frequencies. The detailed distribution of ABO and Rh phenotypes is presented in Table 1.

### 3.3. Documented Vascular Access Thrombosis and ABO Blood Group

A statistically significant association was observed between ABO blood group and documented vascular access thrombosis (*p* = 0.027; Cramer’s V = 0.074). Group A patients exhibited the highest documented thrombosis rate (14.1%), followed by group B (13.8%), group O (10.5%), and group AB (9.7%). When group O was compared with non-O groups combined, documented vascular access thrombosis was less frequent in group O (10.5% vs. 13.3%). Exploratory analysis of the eight ABO–Rh phenotype subgroups revealed a modest association with documented thrombosis (χ^2^ (7) = 16.47, *p* = 0.021; Cramer’s V = 0.074); however, these phenotype-level findings were considered descriptive and were not used as the basis for the main conclusions.

In multivariable binary logistic regression including age, sex, dialysis vintage, diabetes mellitus, vascular access type, and blood group category, the model demonstrated acceptable fit (Hosmer–Lemeshow *p* = 0.235) and was statistically significant overall (*p* < 0.001). Compared with blood group O, non-O blood groups were independently associated with higher odds of documented vascular access thrombosis (OR 1.34, 95% CI 1.06–1.70; *p* = 0.014). Each one-month increase in dialysis vintage was independently associated with a small increase in the odds of documented thrombosis (OR 1.01, 95% CI 1.00–1.01; *p* < 0.001). Age, sex, diabetes mellitus, and vascular access type were not independently associated with documented vascular access thrombosis in the multivariable model (Table 2).

### 3.4. Blood Group-Specific Clinical Characteristics

Comparisons of clinical characteristics across the four major ABO blood groups revealed no significant differences in age (*p* = 0.148), sex distribution (*p* = 0.161), dialysis vintage (*p* = 0.737), unadjusted mortality (*p* = 0.359), or prevalence of diabetes mellitus (*p* = 0.324), hypertension (*p* = 0.422), coronary artery disease (*p* = 0.865), or cerebrovascular disease (*p* = 0.476). Documented vascular access thrombosis differed significantly by ABO group (*p* = 0.027), as did vascular access type distribution (*p* = 0.012). Effect sizes for all categorical comparisons were small (Cramer’s V ≤ 0.074), indicating that absolute differences between groups were limited (Table 3).

### 3.5. Unadjusted Survival Analysis

**Figure 1:**
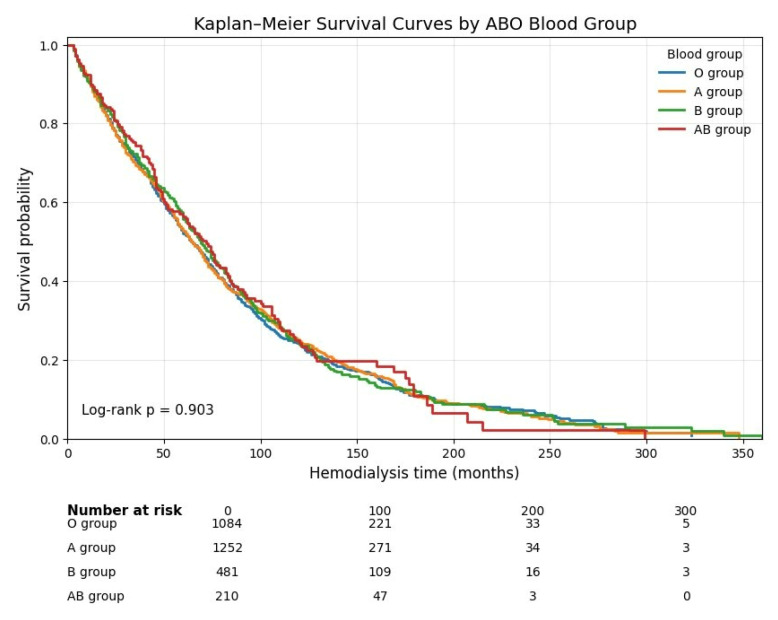
Kaplan–Meier survival curves by ABO blood group with numbers at risk shown below the plot. Unadjusted survival did not differ significantly among the four ABO blood groups (log-rank *p* = 0.903), and blood group phenotype was likewise not independently associated with mortality in multivariable Cox regression adjusted for age, sex, comorbidities, vascular access type, and time-dependent vascular access thrombosis (see Table 4).

Kaplan–Meier survival analysis revealed no statistically significant unadjusted differences in survival among the four ABO blood groups (log-rank *p* = 0.903) (Figure 1). This null result was consistent with the comparable unadjusted mortality proportions across ABO groups observed in Table 3 (*p* = 0.359) and supported the subsequent multivariable analysis that further evaluated the contribution of established demographic factors, comorbidities, and vascular access type to mortality risk.

### 3.6. Multivariable Cox Regression Analysis of Mortality Risk Factors

In the multivariable Cox proportional hazards regression model including age, sex, diabetes mellitus, hypertension, coronary artery disease, vascular access type, blood group phenotype, and time-dependent vascular access thrombosis, several factors were independently associated with all-cause mortality (Table 4). Vascular access thrombosis, modeled as a time-dependent covariate, was independently associated with higher mortality (HR 1.33, 95% CI 1.12–1.59; *p* = 0.002). Each one-year increase in age was associated with a 2% increase in mortality hazard (HR 1.02, 95% CI 1.01–1.02; *p* < 0.001). Female sex was not significantly associated with mortality after adjustment (HR 0.96, 95% CI 0.89–1.05; *p* = 0.449).

Diabetes mellitus (HR 1.49, 95% CI 1.37–1.63; *p* < 0.001), hypertension (HR 1.19, 95% CI 1.09–1.29; *p* < 0.001), and coronary artery disease (HR 1.34, 95% CI 1.21–1.47; *p* < 0.001) were independently associated with increased mortality. Compared with temporary catheter, arteriovenous fistula was associated with substantially lower mortality (HR 0.46, 95% CI 0.37–0.58; *p* < 0.001), whereas permanent catheter use did not reach statistical significance after adjustment (HR 0.79, 95% CI 0.61–1.01; *p* = 0.056).

Blood group phenotype was not independently associated with all-cause mortality. Compared with blood group O, blood groups A (HR 1.01, 95% CI 0.92–1.11; *p* = 0.922), B (HR 0.96, 95% CI 0.85–1.09; *p* = 0.520), and AB (HR 1.03, 95% CI 0.86–1.23; *p* = 0.743) showed no significant association with mortality in the adjusted model. The overall multivariable model was statistically significant (*p* < 0.001).

## 4. Discussion

### 4.1. Blood Group Distribution in Hemodialysis Patients

The blood group distribution observed in our hemodialysis cohort closely mirrors that of the general Turkish population, with group A predominating (41.4%), followed by O (35.8%), B (15.9%), and AB (6.9%). This pattern aligns with previous Turkish epidemiological studies, where group A prevalence of 41.8% and Rh-positive frequencies of 85.0% have been reported [6]. Our finding of 92.7% Rh-positive prevalence is broadly consistent with these national demographic data, reinforcing the representative nature of our study population.

While group O represents the most frequent blood type globally, regional and ethnic variations significantly influence local distributions [7]. The predominance of group A in our Turkish cohort contrasts with some international hemodialysis populations, where varied patterns have been documented. Previous comparative studies examining healthy individuals versus hemodialysis patients have yielded conflicting results regarding blood group distributions, with some reporting no significant differences and others identifying statistical variations in Rh phenotypes [8,9].

### 4.2. Vascular Access Thrombosis and Blood Group

Documented vascular access thrombosis was modestly but significantly more frequent in non-O blood groups (13.3%) than in group O (10.5%), and the non-O blood group remained independently associated with documented thrombosis in multivariable logistic regression (OR 1.34, 95% CI 1.06–1.70; *p* = 0.014) after adjustment for age, sex, dialysis vintage, diabetes mellitus, and vascular access type. This finding is biologically plausible: the ABO gene cluster on chromosome 9q34 influences plasma levels of von Willebrand factor and factor VIII, and non-O individuals exhibit approximately 25% higher plasma concentrations of these procoagulant factors than group O individuals [4]. Large epidemiological studies in the general population have consistently demonstrated higher risks of venous thromboembolism and arterial cardiovascular events in non-O groups [5].

Previous investigations specifically examining vascular access thrombosis in hemodialysis populations have yielded inconsistent results [10]. Our findings extend this literature by demonstrating, in a large Turkish cohort followed across a 15-year period, that the non-O–thrombosis association persists after adjustment for established clinical risk factors and vascular access type. Longer dialysis vintage was also independently associated with documented thrombosis, consistent with cumulative procedural and hemostatic stress on vascular access over time.

It is important to interpret the magnitude of this association cautiously. The absolute difference in documented thrombosis between non-O and O groups was approximately 2.8 percentage points (13.3% vs. 10.5%), corresponding to an odds ratio of 1.34. While statistically significant, the small absolute risk difference limits the standalone clinical utility of blood group as a stratification tool. Blood group is best considered as one of several contributors to vascular access risk awareness rather than a primary clinical decision variable.

### 4.3. Cardiovascular Risk and Comorbidity Associations

The relationship between ABO blood groups and cardiovascular comorbidities in our cohort revealed limited associations. Unlike some previous reports suggesting blood group–specific susceptibility patterns for hypertension and diabetes mellitus [11,12,13,14], we found no significant differences between the four major ABO phenotypes for these conditions. This discrepancy may reflect the complex pathophysiology of end-stage renal disease, where multiple non-genetic factors dominate cardiovascular risk and may obscure blood group–specific effects.

In the broader context of cardiovascular disease, some studies have suggested that end-stage renal disease patients with group O may have protective effects against coronary artery disease severity compared to group A [15]. Our findings do not strongly support these observations in our adjusted analyses, though the increased thrombotic risk in non-O groups may have indirect implications for cardiovascular outcomes.

### 4.4. Mortality Patterns and Time-Dependent Modelling of Thrombosis

Both unadjusted Kaplan–Meier analysis (log-rank *p* = 0.903) and multivariable Cox proportional hazards regression indicated that ABO blood group phenotype was not associated with all-cause mortality in our hemodialysis cohort. Compared with blood group O, the adjusted hazard ratios for blood groups A (HR 1.01, 95% CI 0.92–1.11; *p* = 0.922), B (HR 0.96, 95% CI 0.85–1.09; *p* = 0.520), and AB (HR 1.03, 95% CI 0.86–1.23; *p* = 0.743) all crossed unity with *p*-values exceeding 0.5. The convergence of unadjusted and adjusted analyses provides consistent evidence that blood group phenotype does not independently influence mortality risk in this population and that survival in maintenance hemodialysis is driven predominantly by demographic factors, comorbidities, and vascular access type rather than by ABO phenotype.

Because vascular access thrombosis is an event that occurs during follow-up rather than being present at baseline, we modeled it as a time-dependent covariate in the Cox regression: patients were considered unexposed before the documented month of thrombosis and exposed thereafter. This approach is methodologically appropriate for events ascertained during follow-up and avoids the immortal time bias that can otherwise produce spurious protective associations [16]. Under this time-dependent specification, documented vascular access thrombosis was independently associated with a 33% higher mortality hazard (HR 1.33, 95% CI 1.12–1.59; *p* = 0.002), consistent with clinical expectations and with the established association between vascular access dysfunction and adverse outcomes in hemodialysis patients.

Among traditional risk factors, diabetes mellitus, coronary artery disease, hypertension, and age remained the dominant independent predictors of mortality, with effect sizes consistent with the prior literature in hemodialysis cohorts. Compared with temporary catheter use, arteriovenous fistula was associated with substantially lower mortality (HR 0.46, 95% CI 0.37–0.58; *p* < 0.001), consistent with current guidelines that favor arteriovenous fistula as the preferred vascular access whenever feasible. Permanent (tunnelled) catheter use showed a borderline association (HR 0.79, 95% CI 0.61–1.01; *p* = 0.056), with point estimates intermediate between temporary catheter and arteriovenous fistula.

Mechanistic hypotheses linking ABO antigens to mortality include their role as receptors or ligands for pathogens and their influence on endothelial injury markers. Blood group O individuals appear less susceptible to SARS-CoV-2 infection than non-O groups, while groups A and B show increased COVID-19 risk [17,18]. Blood group A has also been associated with greater endothelial injury and acute kidney injury risk in critically ill patients [19]. In kidney transplant recipients, patient survival has generally not differed between O and non-O groups, although death-censored graft survival has been reported as inferior for AB compared with O recipients [20]. Our adjusted analysis suggests that any such effects, if present in hemodialysis patients, are not sufficient to render blood group phenotype an independent prognostic factor for all-cause mortality after accounting for established cardiovascular risk factors and vascular access type.

### 4.5. Clinical Implications and Future Directions

Our findings indicate that non-O blood group is independently but modestly associated with documented vascular access thrombosis in hemodialysis patients. While statistically significant, the small absolute risk difference (≈ 2.8 percentage points) limits the standalone clinical utility of blood group phenotyping as a primary risk-stratification tool. Blood group may instead contribute incrementally to vascular access risk awareness alongside established and modifiable factors such as dialysis vintage, vascular access type, and comorbidity burden. Decisions about access type, surveillance frequency, and antithrombotic strategies should continue to be driven by these established factors.

With respect to mortality, our adjusted analyses do not support blood group phenotyping as an independent risk-stratification tool. Clinical efforts to reduce mortality should focus on modifiable cardiovascular risk factors (diabetes mellitus, hypertension, coronary artery disease), prevention and timely treatment of vascular access thrombosis, and prioritization of arteriovenous fistula as the preferred access type whenever feasible.

### 4.6. Strengths and Limitations

The strengths of our study include the large sample size (n = 3027), long follow-up period (15 years), inclusion of vascular access type as a covariate in both thrombosis and mortality models, the use of multivariable logistic regression for the thrombosis outcome, and time-dependent Cox modelling for vascular access thrombosis in the mortality analysis. The application of a pre-specified, clinically driven multivariable strategy (rather than *p*-value–based variable selection) reduces the risk of model instability arising from correlated comorbidities.

Several limitations must be acknowledged. The single-center, retrospective design may limit generalizability and introduce potential selection bias. Vascular access thrombosis events were identified retrospectively from documented clinical records rather than through standardized prospective surveillance; therefore, the primary outcome is best interpreted as documented vascular access thrombosis, and the true incidence may be underestimated, particularly for events that did not require intervention. Cause-specific mortality data were not available; all-cause mortality was used as the outcome, which precludes assessment of blood group associations with specific causes of death (e.g., cardiovascular vs. infectious).

Important confounders such as anticoagulant and antiplatelet use, vWF and factor VIII levels, inflammatory markers (e.g., C-reactive protein, neutrophil-to-lymphocyte ratio), dialysis adequacy (Kt/V, urea reduction ratio), nutritional indices, and smoking status were not systematically available in the retrospective dataset and could not be incorporated into the multivariable models. Their absence represents unmeasured confounding for both the thrombosis and mortality analyses. A pre-planned era-stratified sensitivity analysis (e.g., 2010–2017 versus 2018–2025) could not be performed due to limitations in the granularity of the available longitudinal dataset and the absence of consistently coded enrolment-era flags; this represents an additional limitation, and temporal variations in clinical practice across the 15-year study period may influence outcomes independently of blood group.

ABO–Rh phenotype-level analyses were conducted as exploratory and were not adjusted for multiple comparisons; these findings should be interpreted descriptively rather than as confirmatory. Small sample sizes in rare blood group subgroups reduce statistical power and increase uncertainty around effect estimates.

Future research should prioritize multicenter prospective study designs with larger sample sizes, standardized prospective surveillance for incident vascular access thrombosis, incorporation of mechanistic biomarkers (vWF, factor VIII, inflammatory indices), cause-specific mortality ascertainment, and validation in ethnically diverse populations to establish the generalizability of blood group–thrombosis associations across different genetic backgrounds.

## 5. Conclusions

In this analysis of 3027 hemodialysis patients followed across 15 years, non-O blood groups were modestly but independently associated with documented vascular access thrombosis (OR 1.34, 95% CI 1.06–1.70), and documented vascular access thrombosis was independently associated with higher all-cause mortality when modeled as a time-dependent exposure (HR 1.33, 95% CI 1.12–1.59). Blood group phenotype was not independently associated with all-cause mortality after adjustment for established clinical risk factors and vascular access type. Established factors—age, diabetes mellitus, hypertension, coronary artery disease, and arteriovenous fistula as vascular access—remained the principal independent determinants of survival.

Blood group may contribute incrementally to vascular access risk awareness in hemodialysis patients, but the small absolute risk difference limits its standalone clinical utility. Clinical efforts to reduce thrombosis and mortality in this population should continue to focus on modifiable risk factors, optimization of vascular access (favoring arteriovenous fistula), and timely management of access-related complications.

## Figures and Tables

**Table 1:** Prevalence of ABO and Rh blood groups in hemodialysis patients.

	O	A	B	AB	Total
Rh (+)	1009 (93.1)	1161 (92.7)	451 (93.8)	185 (88.1)	2806 (92.7)
Rh (−)	75 (6.9)	91 (7.3)	30 (6.2)	25 (11.9)	221 (7.3)
Total	1084 (35.8)	1252 (41.4)	481 (15.9)	210 (6.9)	3027 (100)

Data are presented as n (%). Percentages within each ABO column (O, A, B, AB) are calculated relative to the column total (e.g., 25/210 = 11.9% of AB patients were Rh-negative). Percentages in the Total row are calculated relative to the entire cohort (e.g., the AB group constitutes 210/3027 = 6.9% of the cohort). When expressed relative to the entire cohort, the AB Rh-negative phenotype represents 25/3027 = 0.83%.

**Table 2:** Univariate and multivariable binary logistic regression analysis of factors associated with documented vascular access thrombosis.

Variable	Univariate OR (95% CI)	*p*-Value	Multivariable OR (95% CI)	*p*-Value
Age (per year)	0.99 (0.98–1.00)	0.116	0.99 (0.99–1.01)	0.391
Sex (Female vs. Male)	0.86 (0.69–1.08)	0.185	0.88 (0.70–1.09)	0.250
Dialysis vintage (per month)	1.01 (1.00–1.01)	<0.001	1.01 (1.00–1.01)	<0.001
Diabetes mellitus (Yes vs. No)	0.95 (0.77–1.18)	0.658	0.92 (0.73–1.15)	0.456
Vascular access type (vs temporary catheter)				
Arteriovenous fistula	1.02 (0.55–1.89)	0.944	0.81 (0.43–1.52)	0.515
Permanent (tunnelled) catheter	0.82 (0.41–1.62)	0.561	0.78 (0.39–1.57)	0.483
Blood group (non-O vs. O)	1.34 (1.06–1.69)	0.014	1.34 (1.06–1.70)	0.014

CI, confidence interval; OR, odds ratio. Reference categories: male sex; absence of diabetes mellitus; temporary catheter for vascular access type; blood group O. Multivariable model: Hosmer–Lemeshow *p* = 0.235; overall model *p* < 0.001.

**Table 3:** Clinical characteristics and documented vascular access thrombosis by ABO blood group.

Variable	Total (*n* = 3027)	O (*n* = 1084)	A (*n* = 1252)	B (*n* = 481)	AB (*n* = 210)	Effect Size	*p*-Value
Age, years, median (range)	66 (18–90)	66 (18–90)	65 (18–90)	66 (18–90)	66 (19–90)	ε^2^ < 0.001	0.148 ^a^
mean ± SD	63.95 ± 13.74	64.42 ± 13.79	63.41 ± 13.64	64.38 ± 13.99	63.68 ± 13.42		
Sex, *n* (%)						0.047	0.161 ^b^
Men	1766 (58.3)	639 (58.9)	725 (57.9)	293 (60.9)	109 (51.9)		
Women	1261 (41.7)	445 (41.1)	527 (42.1)	188 (39.1)	101 (48.1)		
Dialysis vintage, months	53 (3–360)	53 (3–331)	52 (3–348)	56 (3–360)	51.5 (3–299)	ε^2^ < 0.001	0.737 ^a^
mean ± SD	67.07 ± 54.80	66.79 ± 55.22	66.48 ± 54.29	68.69 ± 57.14	65.97 ± 50.12		
Mortality, *n* (%)						0.044	0.359 ^b^
Deceased	2260 (74.7)	816 (75.3)	928 (74.1)	368 (76.5)	148 (70.5)		
Alive	767 (25.3)	268 (24.7)	324 (25.9)	113 (23.5)	62 (29.5)		
Documented thrombosis, *n* (%)	373 (12.3)	112 (10.5)	175 (14.1)	66 (13.8)	20 (9.7)	0.074	0.027 ^b^
Diabetes mellitus, *n* (%)	1647 (54.9)	574 (53.6)	677 (54.4)	276 (57.9)	120 (58.3)	0.067	0.324 ^b^
Cerebrovascular disease, *n* (%)	231 (7.7)	91 (8.5)	91 (7.3)	31 (6.5)	18 (8.7)	0.048	0.476 ^b^
Hypertension, *n* (%)	1588 (53.0)	563 (52.6)	663 (53.3)	243 (50.9)	119 (57.8)	0.054	0.422 ^b^
Coronary artery disease, *n* (%)	659 (21.8)	237 (22.1)	279 (22.4)	98 (20.5)	45 (21.8)	0.037	0.865 ^b^
Vascular access type, *n* (%)						0.063	0.012 ^b^
Arteriovenous fistula	2555 (84.4)	903 (83.3)	1042 (83.2)	417 (86.7)	193 (91.9)		
Permanent (tunnelled) catheter	376 (12.4)	140 (12.9)	172 (13.7)	48 (10.0)	16 (7.6)		
Temporary catheter	96 (3.2)	41 (3.8)	38 (3.0)	16 (3.3)	1 (0.5)		

Data are presented as median (minimum–maximum), mean ± standard deviation, or n (%). ^a^ Kruskal–Wallis test; ^b^ Pearson chi-square test or Fisher’s exact test, as appropriate. Effect sizes are reported as Cramer’s V for categorical variables and epsilon-squared (ε^2^) for continuous variables compared using the Kruskal–Wallis test. All effect sizes indicated negligible to small associations. SD, standard deviation.

**Table 4:** Univariate and multivariable Cox proportional hazards regression analysis of mortality risk factors, with vascular access thrombosis included as a time-dependent covariate.

Variable	Univariate HR (95% CI)	*p*-Value	Multivariable HR (95% CI)	*p*-Value
Vascular access thrombosis (time-dependent)	1.26 (1.06–1.51)	0.009	1.33 (1.12–1.59)	0.002
Age (per year)	1.02 (1.01–1.02)	<0.001	1.02 (1.01–1.02)	<0.001
Sex (Female vs. Male)	0.90 (0.83–0.98)	0.014	0.96 (0.89–1.05)	0.449
Diabetes mellitus (Yes vs. No)	1.72 (1.58–1.87)	<0.001	1.49 (1.37–1.63)	<0.001
Hypertension (Yes vs. No)	1.39 (1.28–1.51)	<0.001	1.19 (1.09–1.29)	<0.001
Coronary artery disease (Yes vs. No)	1.50 (1.37–1.65)	<0.001	1.34 (1.21–1.47)	<0.001
Vascular access type (vs temporary catheter)				
Arteriovenous fistula	0.47 (0.38–0.59)	<0.001	0.46 (0.37–0.58)	<0.001
Permanent (tunnelled) catheter	0.89 (0.69–1.14)	0.348	0.79 (0.61–1.01)	0.056
Blood group (vs O)				
A	0.99 (0.90–1.09)	0.820	1.01 (0.92–1.11)	0.922
B	0.95 (0.84–1.08)	0.436	0.96 (0.85–1.09)	0.520
AB	0.96 (0.81–1.14)	0.645	1.03 (0.86–1.23)	0.743

CI, confidence interval; HR, hazard ratio. Reference categories: male sex; absence of diabetes mellitus; absence of hypertension; absence of coronary artery disease; temporary catheter for vascular access type; blood group O. Vascular access thrombosis was modeled as a time-dependent covariate: patients were considered unexposed before the documented month of thrombosis and exposed thereafter. Overall multivariable model *p* < 0.001.

## Data Availability

The datasets used and/or analyzed during the current study are available from the corresponding author on reasonable request. The data are not publicly available due to privacy and ethical restrictions.

## Abbreviations

The following abbreviations are used in this manuscript:

ABO	ABO blood group system
AB	Blood group AB
CI	Confidence interval
ESRD	End-stage renal disease
HR	Hazard ratio
OR	Odds ratio
Rh	Rhesus blood group system
SARS-CoV-2	Severe acute respiratory syndrome coronavirus 2
SPSS	Statistical Package for the Social Sciences
vWF	von Willebrand factor
